# How does ageing affect grasp adaptation to a visual–haptic size conflict?

**DOI:** 10.1007/s00221-018-5288-1

**Published:** 2018-05-23

**Authors:** Samuel Couth, Emma Gowen, Ellen Poliakoff

**Affiliations:** 1 Division of Human Communication, Development and Hearing, School of Health Sciences, Faculty of Biology, Medicine and Health, The University of Manchester, A3.16 Ellen Wilkinson Building, Oxford Road, M13 9PL Manchester, UK; 2 Division of Neuroscience and Experimental Psychology, School of Biological Sciences, Faculty of Biology, Medicine and Health, The University of Manchester, Zochonis Building, Oxford Road, M13 9PL Manchester, UK

**Keywords:** Ageing, Grasp adaptation, Haptics, Motor control

## Abstract

Previous research suggests that the ability to adapt motor behaviour to sudden environmental changes may be impaired in older adults. Here, we investigated whether the adaptation of grasping behaviour in response to a visual–haptic size conflict is also affected by increasing age. 30 older and 18 young adults were instructed to grasp a hidden block whilst viewing a second block in a congruent position. Initially block sizes were equal, but after a set number of trials a sensory conflict was introduced by covertly changing the hidden block for a smaller or larger block. The scale and speed of maximum grasp aperture adaptation to the increase or decrease in the size of the hidden block was measured. Older adults successfully adapted to the visual–haptic size conflict in a similar manner to young adults, despite a tendency to adapt less when the hidden block increased in size. This finding is attributed to the physical capabilities of the grasping hand of older adults, rather than an effect of age-related sensory or cognitive decline. The speed of grasp adaptation did not differ between age groups; however, awareness of the visual–haptic conflict lead to faster adaptation. These findings suggest that sensorimotor adaptation for grasping is intact for cognitively healthy older adults.

## Introduction

To effectively coordinate movement in an ever-changing environment, the central nervous system must predict the sensory consequences of performing an action (e.g. vision, touch and proprioception) and compare them to the actual sensory consequences of performing an action, and then use this information to adapt behaviour accordingly. Such a system has been termed an internal forward model of action (Kawato and Wolpert 1998; Wolpert et al. 1998; Wolpert and Kawato 1998; Blakemore et al. 1998; Wolpert and Flanagan 2001). Specifically, predicted sensorimotor outcomes [or representations, see Jeannerod (1984, 1994a, b)] are generated—and continually updated—by previous experience of performing an action. Discrepancies between predicted and actual sensory outcomes of performing an action lead to a recalibrated representation of the action, which is forwarded to the motor system. This process is repeated until the predicted and actual sensory outcomes are aligned (i.e. the motor behaviour has fully adapted). In addition, strategic control mechanisms including visual guidance, error-corrective (on-line) feedback and cognitive mechanisms, such as spatial working memory, may contribute to this adaptation process (Redding and Wallace 1996; Anguera et al. 2011; Langan and Seidler 2011).

A common way to assess motor adaptation is to introduce a conflict between sensory cues and measure the extent and rate of behaviour change to resolve the incongruity between predicted and actual sensory outcomes. For example, prism glasses have been used to displace visual cues whilst participants perform manual aiming movements [for review see Welch (1986)]. The introduction of the prisms causes the aim to be misplaced (usually in the direction of the visual distortion). The sensorimotor system then iteratively recalibrates the aim until the sensorimotor mismatch is realigned and precision aiming has returned. On removal of the prism glasses, it is common to see a post-exposure effect, known as an ‘aftereffect’, whereby manual aiming movements are overcompensated in the opposite direction to the visual displacement. These aftereffects gradually decay and are thought to represent a re-adaptation period where sensorimotor cues must be realigned once more. The strength of aftereffects (i.e. time taken to re-adapt) may be positively correlated with the initial amount of adaptation that occurred, and so has been used as an indicator of the strength of adaptation (Fernández-Ruiz and Díaz 1999). While adaptation may rely on both internal models and strategic control mechanisms, this re-adaptation is almost exclusively governed by internal models to correct the movement, since strategic control mechanisms dissipate quickly in the absence of a sensorimotor discrepancy (Redding and Wallace 1996; McNay and Willingham 1998; Bock 2005; Bock and Girgenrath 2006; Cressman et al. 2010). Similar to these traditional models of adaptation, it has also been shown that the rate of motor adaptation can be predicted using a Bayesian learner model which optimally combines knowledge and belief (i.e. uncertainty) about the properties of the motor system with new knowledge gathered through repeated exposure to a sensory disturbance (Kording et al. 2007).

In the current work, we were interested in understanding how sensorimotor adaptation is affected in older adults. It is well documented that movement accuracy and speed declines with increasing age (Ward and Frackowiak 2003; Seidler et al. 2010; Leversen et al. 2012; Morrison and Newell 2012). These impairments in motor performance may be associated with structural and physiological changes in the ageing brain, such as reduced volume to prefrontal, striatal and cerebellar areas (Scahill et al. 2003; Raz et al. 2005; Peters 2006) and altered neurotransmitter dynamics (Mora et al. 2007), as well as ageing of peripheral sensory organs (Nusbaum 1999) and effector limbs (i.e. muscles, joints, bones etc., Faulkner et al. 2007; Loeser 2010). However, the extent to which impaired sensorimotor adaptation may also contribute to poorer movement performance in older adults is less certain.

A number of studies using visual distortions (i.e. via prism glasses or screen rotations) have demonstrated slower and/or reduced adaptation in older adults during exposure to the sensory discrepancy (McNay and Willingham 1998; Fernández-Ruiz et al. 2000; Buch et al. 2003; Bock 2005; Bock and Girgenrath 2006), whereas others have shown similar levels and rates of adaptation for young and older adults (Canavan et al. 1990; Etnier and Landers 1998; Roller et al. 2002; Buch et al. 2003; Cressman et al. 2010). A consistent finding, however, is that aftereffects are not reduced with ageing, and may even be larger in older adults (for review of motor adaptation in ageing, see King et al. 2013). Accordingly, it has been proposed that cognitive processes (i.e. strategic control mechanisms) used to resolve the sensory conflict may decay with age, but internal models employed post-exposure to the sensory conflict may be resistant to ageing (Bock and Schneider 2002; Bock 2005; Bock and Girgenrath 2006; Heuer and Hegele 2008; Hegele and Heuer 2010; Anguera et al. 2011; Langan and Seidler 2011; Huang et al. 2017). In support, Buch et al. (2003) showed similar adaptation for young and older adults when the sensory conflict was introduced gradually compared to an abrupt change (cf. Bock and Girgenrath 2006). Since participants were less aware of the gradual sensory mismatch, it was suggested that fewer learning strategies were required to solve the discrepancy, and so adaptation was less susceptible to age-related cognitive decline. Similarly, Huang et al. (2017) showed that the adaptation of saccadic eye movements on a simple tracking task was similar for young and older adults, however adaptation was less pronounced for older adults when required to make a perceptual judgement about the target of the saccade; a process that requires executive strategies which are also more vulnerable to age-related cognitive decline. Furthermore, it has been demonstrated that, in line with the Bayesian learner model (Kording et al. 2007), older adults’ predictions of the environment (i.e. internal models) are refined and optimised throughout the lifespan as a result of repeated experience (Moran et al. 2014).

An alternative explanation for the similar levels of adaptation observed in young and older adults is that previous studies have principally involved relatively simple manual aiming-type tasks, and therefore the adaptive processes involved could be resilient to the effects of ageing. In support, Bruijn et al. (2012) demonstrated reduced aftereffects in older adults on a locomotor adaptation task, where the recruitment of multiple body segments for gait adaptation is more operationally complex. However, it has been proposed that gait and posture control are reliant on anterior cerebellar regions which show substantial volume reduction with increasing age (Andersen et al. 2003). Therefore, it is difficult to disentangle the effects of task complexity from the effects of neurodegeneration on locomotor adaptation in older adults. On the contrary, visuomotor adaptation for manual aiming-type tasks is reliant on posterior cerebellar regions, which are less affected by ageing (Krakauer et al. 2004; King et al. 2013), and so by employing a task which uses similar brain regions to manual aiming, but which is also more operationally complex, it might be possible to elucidate the effects of ageing on visuomotor adaptation. For example, reaching and grasping is a relatively complex behaviour since it implies further action to achieve a goal compared to manual aiming alone (e.g. Carnahan et al. 1993), and yet the neural circuits for reaching and grasping and for manual aiming have been shown to have widespread, overlapping activations [for review, see Filimon (2010)].

Previous research has explored how young adults adapt prehensile action following the introduction of a multisensory conflict. For example, grasping kinematics have been measured during illusions that distort haptic object size (Gentilucci et al. 1995; Säfström and Edin 2004, 2005, 2008), orientation (Weigelt and Bock 2007, 2010) and distance (Coats et al. 2008) properties relative to the visual properties of the “same” object. Säfstrӧm and Edin (2004) measured maximum grasp aperture (MGA) of the hand reaching towards an unseen ‘haptic’ block, whilst viewing a mirror-reflected image of a ‘visual’ block [see also, Weigelt and Bock (2007)]. The position of the mirror-reflected visual block corresponded to the position of the unseen haptic block, thus appearing to be the same object, whilst the mirror also blocked visual feedback of the grasping hand. Initially, visual and haptic blocks were the same physical size, but after a set number of trials a sensory conflict was introduced by replacing the unseen haptic block with a smaller or larger block. Over time, all participants adjusted their MGA to the size of the smaller/larger haptic block, even if they were unaware of the abrupt change.

Previous research investigating grasping behaviour more broadly in older adults has demonstrated poorer precision dexterity, slower arm and hand movements and weaker grip-strength [for reviews see Carmeli et al. (2003, Morrison and Newell 2012)]. What remains unknown, however, is whether older adults also display altered grasp adaptation, which could be detrimental to prehensile coordination. Given that much of our interaction with the environment involves reaching and grasping, it is likely that any age-related effects would have a strong impact on activities of daily living.

In the current work, we use a similar paradigm to Säfstrӧm and Edin (2004) to explore how older adults adapt relatively complex grasping behaviour in response to an abrupt conflict in visual and haptic cues. By introducing an abrupt haptic size change, it was expected that this would be more cognitively demanding for older adults, and thus the scale of MGA adaptation would be less and slower to appear (Bock and Girgenrath 2006). Moreover, by introducing a period of re-adaptation where participants were required to readjust their grasp size back to equal visual–haptic block sizes, it was possible to monitor the strength of aftereffects. It was predicted that aftereffects would be similar for both young and older adults, since this period is only reliant on sensory recalibration (internal model) processes, which have been found to be robust to the effects of ageing (Bock 2005; Bock and Girgenrath 2006).

## Methods

### Participants

30 older adults (mean age = 73.2 ± 6.1 years; female *n* = 16; right handed *n* = 27) and 18 young adults (mean age = 22.2 ± 6.4 years; female *n* = 11; right handed *n* = 15) took part in this experiment. Young adults consisted of psychology undergraduate students and other volunteers recruited via the University of Manchester’s research volunteering website. Older adults were recruited via local Manchester community groups and advertisements displayed in local newspapers and council webpages. All participants were required to have equal to or better than 6/12 (20/40) visual acuity (with or without correction) as assessed via the Snellen letter chart. Older participants were screened for dementia using the Mini-Mental State Examination (MMSE, score ≥ 24 indicates normal cognition; Folstein et al. 1975). Participants had no history of any other neurological conditions (e.g. Parkinson’s disease or neuropathy) or significant head injuries. The study was approved by the University of Manchester Research Ethics Committee in accordance with the Declaration of Helsinki 1964, and written informed consent was obtained from all participants.

### Stimuli and apparatus

**Fig. 1:**
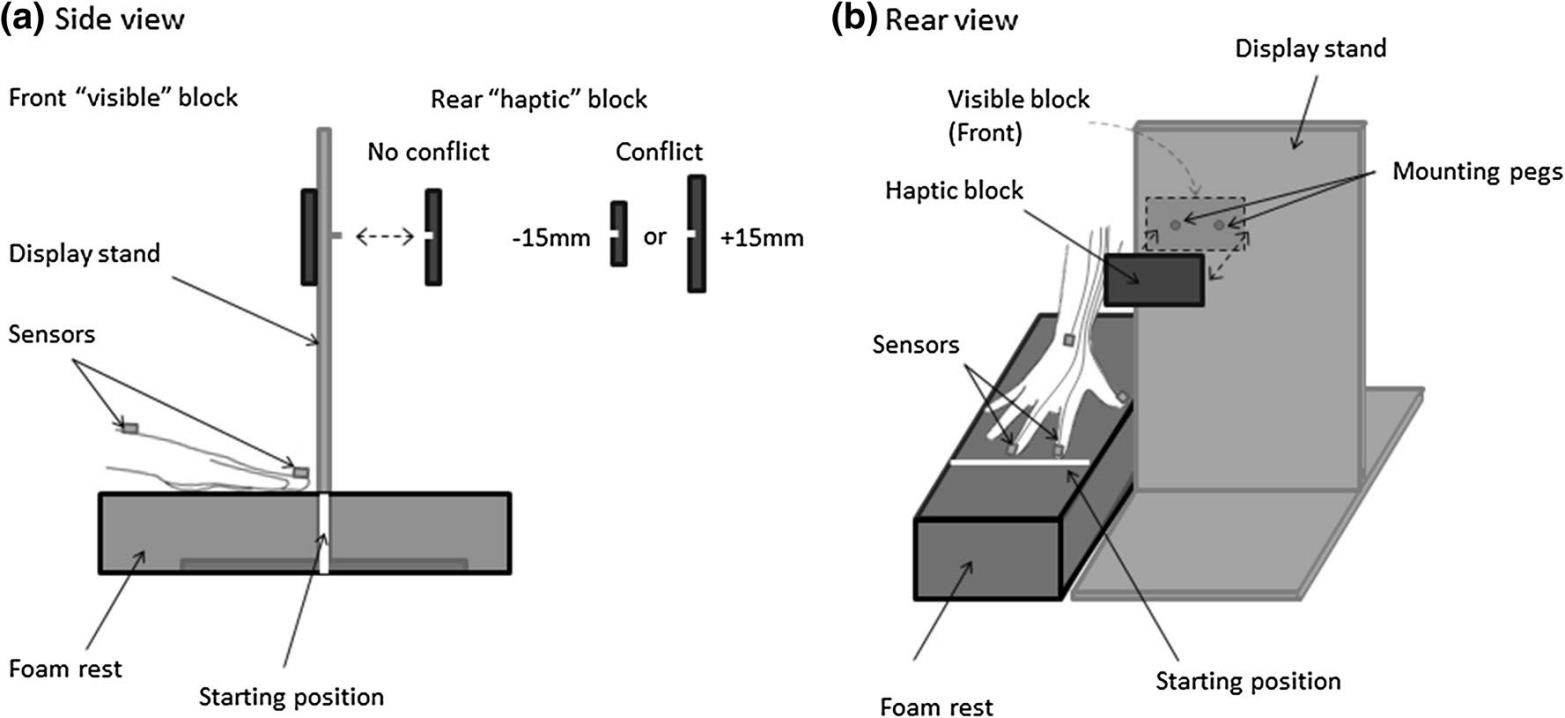
Apparatus and stimuli shown from **a** side view and **b** rear view. The setup was reversed for left-handed participants

Stimuli consisted of eight wooden blocks which were all equal in width (100 mm) and depth (10 mm), but had variable height ranging from 25 to 85 mm in 15 mm increments. There were two blocks of sizes 40, 55 and 70 mm, and one of sizes 25 and 85 mm. An opaque acrylic display stand was used to mount blocks on to the front (visible block) and back (haptic block). The centre of the blocks was 280 mm from the surface of the desk and 110 mm from the top of the display stand. The wooden blocks could be detached/fixed via two acrylic pegs attached to the display stand (Fig. 1a).

Participants wore PLATO visual occlusion spectacles (Translucent Technologies Inc.) throughout the experiment. MatLab (Mathworks, Inc.) was used to trigger the spectacles to become transparent, thus allowing precise timing of visual input (~ 4 ms lag time).

Polhemus LIBERTY motion tracking equipment and MotionMonitor software (Innovative Sports Training, Inc.) were used to collect movement data from participants in the *x*—(left to right), *y*—(front to back) and *z*—(up and down) planes. Four sensors were attached to the participant’s dominant hand, secured to the tip of the nail of the index finger, middle finger and thumb, plus the top of the wrist (Fig. 1b). Each sensor was sampled at 120 Hz. Movement recording began when the PLATO spectacles turned transparent.

### Procedure

Prior to starting the task, the experimenter showed the participant the stimuli and demonstrated the task procedure. Participants were explicitly told that the blocks were the same on the front and back, and appeared as one single stimulus.

Participants were seated in a well-lit room facing the display stand. The seat height was adjusted so that eye level was approximately aligned with the centre of where visible blocks would be presented. Participants placed their dominant hand out in front of them on a foam rest, with their hand flat and facing downwards and behind a white line aligned with the edge of the display stand (Fig. 1b). This indicated the start and end positions of the hand. Participants sat at a distance which enabled a comfortable reach around the display stand to where the non-visible (haptic) block was positioned. Participants were instructed to keep their arm out in this position throughout the experiment to ensure that their distance from the stimuli remained constant.

At the start of the experiment, the PLATO spectacles occluded the participant’s vision. The experimenter attached equal-sized blocks to the front and back of the display stand (40, 55 or 70 mm), before manually initiating the first trial. Commencement of a trial caused the PLATO spectacles to become transparent, signalling that the participant should observe the visible block presented at the front of the display stand and to make a reach and grasp action of the haptic block at the rear of the display stand. The participant was instructed to grasp the haptic block with their thumb on the top and index finger on the bottom, hold the block for ~ 1 s, and then return their hand to the start position. Vision of the grasping hand was occluded by the display stand. The PLATO spectacles remained transparent for 4 s in total before turning opaque, allowing the participant time to complete the reach and grasp action. The PLATO spectacles remained opaque for a further 4 s (inter-trial interval) before the next trial commenced automatically. Participants were instructed to repeat the reach and grasp action every time that the spectacles turned transparent.

After the PLATO spectacles had turned opaque on the 11th trial, the experimenter removed the haptic block at the rear of the display stand and replaced with a different block which was either 15 mm shorter or taller than the visible block (i.e. 7.5 mm shorter/taller at the top and bottom). Participants continued to grasp this shorter/taller haptic block for a further 23 trials (i.e. the adaptation period). After the PLATO spectacles had turned opaque on the 34th trial, the experimenter removed the shorter/taller haptic block, and replaced with the original haptic block which is equal in size to the visible block. Participants continued to grasp the equal-sized haptic block for a further 16 trials (50 trials total). These final 16 trials reflect a re-adaptation period whilst the MGA readjusts back to its pre-adapted state; the length of which can be used to determine the strength of aftereffects (Säfström and Edin 2004, 2008).

Participants completed 6 blocks of trials (300 trials total); 3 visible block sizes (40, 55, 70 mm), with haptic blocks either increasing or decreasing in size (15 mm taller/shorter). The order of blocks of trials was randomised between participants.

At the end of the experiment, the experimenter attempted to determine whether the participant was explicitly aware of the visual–haptic size conflict. Participants were asked to describe the apparatus and procedure, and also asked what they thought the purpose of the experiment was. Occasions where participants explicitly commented on the visual–haptic size conflict during the experiment were noted by the experimenter.

To prevent participants from noticing the visual–haptic manipulation, and to prevent participants who were aware of the manipulation from anticipating a change in the haptic block size, the experimenter would occasionally remove and replace the same haptic block from the display stand (approximately once every 5–10 trials). Therefore any other cues (i.e. auditory) could not be used to reliably predict when the haptic block size had changed.

### Data analysis

There are many kinematic variables and forces/moments that could be analysed in a reaching and grasping task, and there is evidence to suggest that movement speed and grasp aperture pre-shaping is affected by the size of the target object (e.g. Jeannerod 1984; Marteniuk et al. 1987). However, given that the visual properties of the object were unaltered and that the change in the haptic block size was relatively quite small (15 mm), we focussed on MGA only, since this is a clearly identifiable kinematic event which directly reflects the relationship between actual and perceived object sizes (Jeannerod 1984; Marteniuk et al. 1990). MGA was calculated as the maximum distance in 3D space between the index finger and thumb sensors minus the distance between the sensors when the index finger and thumb when lightly pinched together.

The first two trials of each experimental block were discounted as practise trials. Occasions where participants failed to respond or where the whole movement was not captured within the 4 s movement period were discounted as errors. Given the task simplicity, error rate was low with response accuracy at 98 and 99% for older and young adults, respectively. As such, no further analysis of error rate was conducted.

#### Determining the scale of grasp adaptation

For each participant, the average MGA for the first nine trials (prior to introducing the visual–haptic size conflict) was calculated for each visible block size (40, 55 and 70 mm) collapsed across both conditions where the haptic block later increased/decreased in size. A linear regression line (*fV*) was then fitted to determine the relationship between the size of the visible block size (*V;* 40, 55 or 70 mm) and the MGA for each participant.

**Fig. 2:**
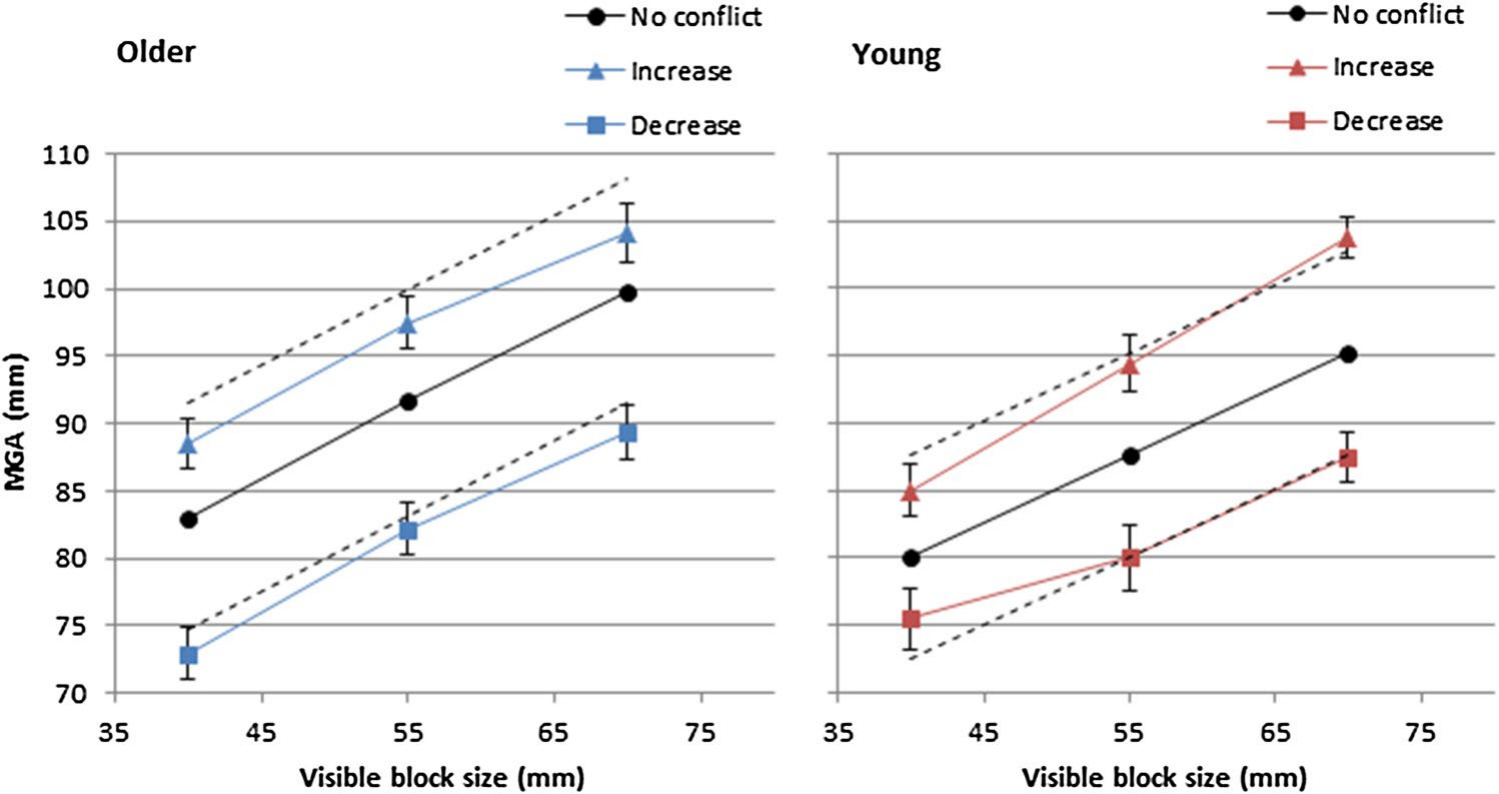
Solid lines represent mean maximum grasp aperture (± SEM) during equal visual–haptic conditions (circles), increased haptic size conditions (triangles), and decreased haptic size conditions (squares). Dashed lines represent full haptic adaptation (i.e. *f*(*V* + *Δ*); Eq. 1). Older adults left panel (blue), young adults right panel (red). Colour version available online

Following the introduction of the visual–haptic size conflict, the participant adjusted their MGA (either knowingly or unknowingly) to reflect the change in haptic block size. The average of the last four trials during the visual–haptic conflict (trials 30–34) was taken as the fully adapted maximum grasp aperture (aMGA; no further adaptation occurs after 20 trials; Säfström and Edin 2004). aMGA was then used to calculate the scale of grasp adaptation to the increase/decrease size of the haptic block:

1 $${\text{Adaptation~}}\left( V \right)\;=\;~\left( {\frac{{{\text{aMGA}} - fV}}{{f\left( {V+\Delta } \right) - fV}}} \right),$$

where *Δ* represents the increase or decrease in haptic block size (± 15 mm). Therefore, *f*(*V* + *Δ*) was calculated by taking the size of the visual block (*V*; 40, 55 or 70) ± the size change of haptic block (15 mm) and multiplying by the slope of the regression line, and thus represents the change in grasp aperture required to have fully adapted to the size change of the haptic block (see dashed lines in Fig. 2).

A score of 0 suggests that no grasp adaptation occurred after introducing the visual–haptic conflict and a score of 1 suggests full grasp adaptation. The scale of adaptation is, therefore, limited to a minimum of 0 and a maximum of 1 (Eq. 1 adapted from Säfström and Edin 2004).

It was possible that due to physical constraints on grasp size, the ability to change MGA was less when the haptic block increased in size in conflict trials (especially in the 70 mm visible block condition) compared to decreased, which could give the impression that the scale of adaptation was reduced for the larger visible block sizes in increased haptic conflict trials. To assess this possibility, we compared average regression slopes across the visible block sizes for increased, decreased and no-conflict conditions.

#### Rate of grasp adaptation

To measure the rate of adaptation for each participant, the MGA for each trial after the introduction of a shorter/taller haptic block was subtracted from the mean MGA for the first nine trials (equal visual–haptic block size) collapsed across both conditions where the haptic block later increased/decreased in size. This was calculated for each visible block size (40, 55, 70 mm), for both increased and decreased haptic size conditions. An exponential function was then fitted by non-linear regression to the difference in MGA (dMGA) for each visible block size (40, 55, 70 mm), for both increased and decreased haptic size conditions:

2 $${\text{dMGA}}\;=\;a~\; \times \;~{{\text{e}}^{\left( { - b~ \times ~T} \right)}}+c,$$

where *a, b* and *c* are constants, and *T* is the trial number with *T* = 0 corresponding to the first trial with a change in the haptic block size.

The number of trials to reach 50% of the fully adapted state (*T*_50%_) was used to calculate the rate of adaptation:

3 $${T_{50\% }}=\frac{{ - \ln \left( {0.5} \right)}}{b}.$$

#### Rate of re-adaptation

The rate of re-adaptation was calculated and analysed using the same method as for the rate of adaptation. To determine the relationship between the strength of adaptation and aftereffects, correlations were performed between the scale of adaptation and rate of re-adaptation in increased and decreased conditions, for each age group separately.

## Results

### Scale of grasp adaptation

After the introduction of the visual–haptic size conflict, participants adjusted the size of their MGA in the same direction as the change in haptic block size (increased/decreased). The number of participants who noticed this manipulation was 17/30 and 14/18 for older and young groups, respectively. A 2 × 2 chi-square test indicated that there was no significant difference between the proportion of participants who noticed the manipulation between the age groups [*χ*^2^ (1, *n* = 48) = 2.192, *p* = 0.139].

A 3 (visible block size; 40, 55, 70 mm) × 3 (haptic size change; increased, decreased, no-conflict) repeated measures ANOVA, with age group and whether participants noticed the manipulation as between subjects factors, revealed a main effect of visible block size on MGA [*F*(1.469, 64.615) = 149.731, *p* < 0.001, *η*^2^ = 0.773]. As the size of the visible blocks increased, MGA also increased, with pairwise comparisons showing a greater MGA for the 70 mm condition (mean = 97.29 mm) than 55 mm (mean = 89.47 mm), which was in turn larger than 40 mm condition (mean = 81.54 mm) (all *p* < 0.001). There was also a main effect of haptic size change [*F*(1.491, 65.593) = 147.507, *p* < 0.001, *ƞ*^2^ = 0.770], with increased size conditions producing a larger adapted MGA (mean = 95.93 mm) compared to equal size conditions (mean = 90.12 mm), which was in turn larger than decreased size conditions (mean = 82.257 mm) (all *p* < 0.001). No further main effects were observed, although the interaction between haptic size change and age group approached significance [*F*(1.491, 65.593) = 3.332, *p* = 0.055, *ƞ*^2^ = 0.070]. To determine the relative strength of evidence to support the null (i.e. no interaction present) and alternative (i.e. interaction present) hypotheses, a Bayesian repeated measures ANOVA with default priors was conducted using JASP (JASP Team 2016). A Bayes factor (BF) > 3 is considered as substantial evidence for the alternative hypothesis, whereas a BF < 1/3 is considered as substantial evidence for the null (Jeffreys 1961). For the haptic size change and age group interaction, the inclusion Bayes factor across matched models = 1.60, meaning that there was only weak/anecdotal evidence to support the alternative hypothesis. No further interactions were observed (all *p* > 0.05).

**Fig. 3:**
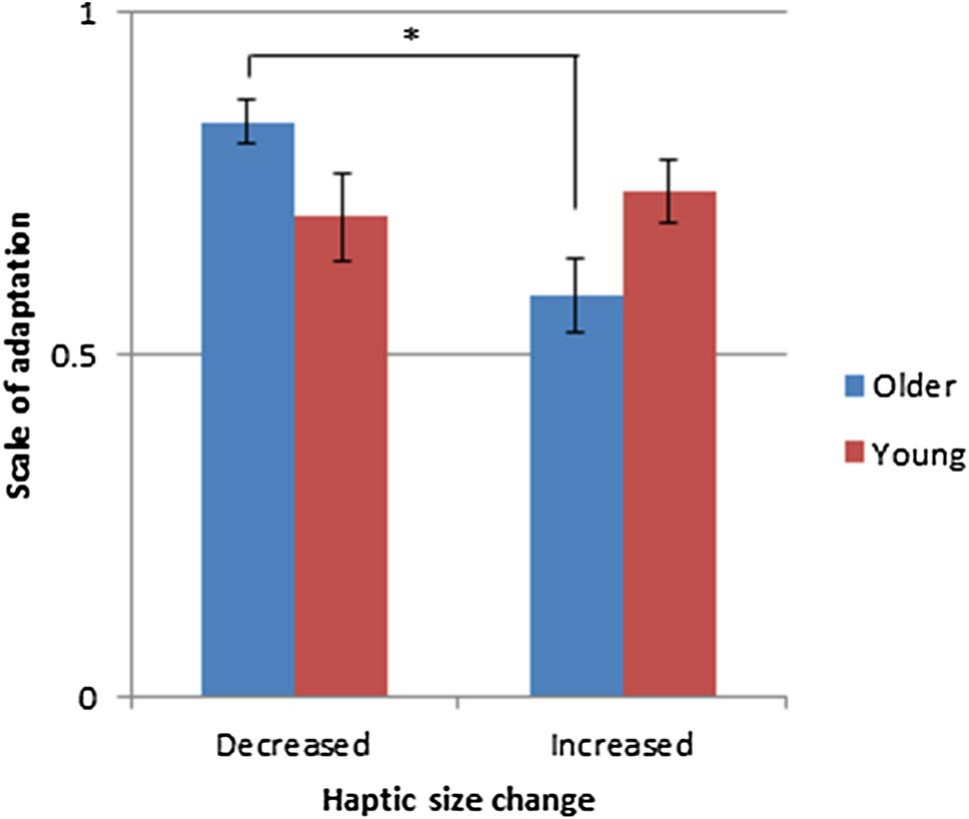
Mean scale of grasp adaptation collapsed over increased and decreased haptic size conflict conditions for older (blue bars) and young (red bars) adults. Asterisks represent a significant difference between increased and decreased conditions (all *p* < 0.008, Bonferroni correction). Colour version available online

A 3 (visible block size; 40, 55, 70 mm) × 2 (haptic size change; increased, decreased) repeated measures ANOVA, with age group and whether participants noticed the manipulation as between subjects factors, revealed no significant main effects on the scale of adaptation (all *p* > 0.05). A significant interaction was found between age group and haptic size change [*F*(1, 44) = 5.744, *p* = 0.021, *ƞ*^2^ = 0.115]. Paired sample *t* tests showed significantly greater adaptation in decreased compared to increased haptic size conditions for older adults [*t*(29) = 3.621, *p* = 0.001, *d* = 1.057]. In contrast, there was no significant difference in adaptation between increased and decreased haptic size blocks for young adults [*t*(17) = − 0.444, *p* = 0.663, *d* = 0.151] (Fig. 3). Comparisons between the age groups showed that there were no significant differences in the scale of grasp adaptation for either increased or decreased haptic size conditions (both *p* > 0.006, Bonferroni correction), but the difference between increased and decreased size conditions was borderline significantly greater for older compared to young adults [*t*(46) = 2.632, *p* = 0.012, *d* = 0.796]. A Bayesian independent sample *t*-test revealed Bayes factor = 4.371, meaning that the alternative hypothesis (i.e. that the mean difference between increased and decreased conditions for older adults was drawn from a different distribution to the young adults) was 4.371 times more likely than the null hypothesis, given the data.

To assess the possibility that adaptation might appear reduced in increased haptic size conditions due to the physical limits of the grasp aperture, a further 3 (visible block size; 40, 55, 70 mm) × 3 (haptic size change; increased, decreased, no-conflict) repeated measures ANOVA was conducted to compare average regression slopes, with age group and awareness of the manipulation as between subject factors. There were no significant main effects or interactions (all *p* > 0.05), thus the relative grasp size to clear the edges of the block remained equivalent for each visible block size, irrespective of whether the haptic block increased or decreased in size, and irrespective of age group.

### Rate of grasp adaptation

**Fig. 4:**
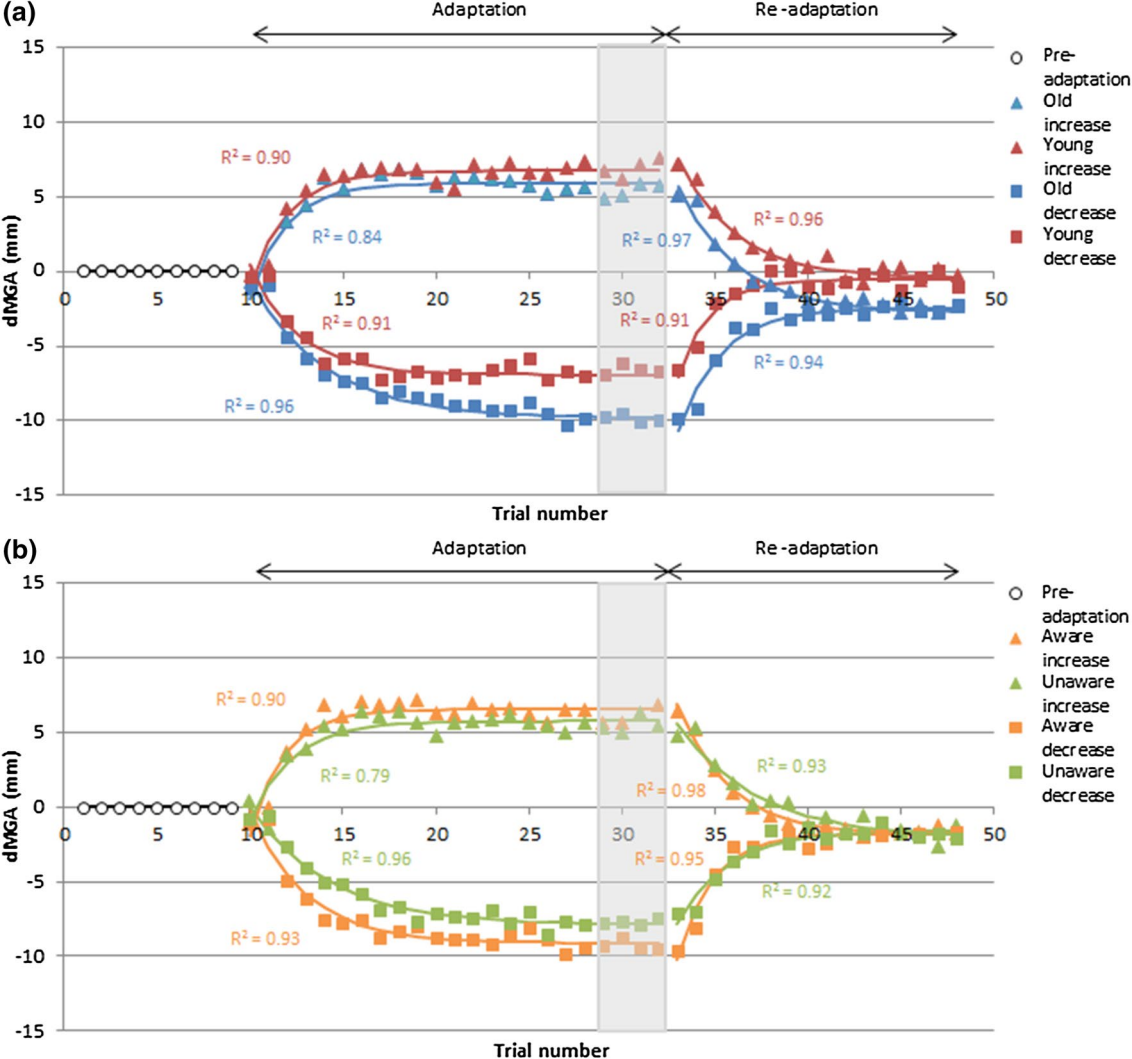
Rate of adaptation split by **a** age group (blue lines older, red lines young) and **b** awareness of the haptic size change (orange lines aware, green lines unaware). dMGA is the difference in grasp size before and after adaptation/re-adaptation (Eq. 2). Triangles represent increased haptic conditions, squares represent decreased haptic conditions and the white circles represent the pre-adaptation period when visual and haptic blocks were equal in size. Grey shaded areas show the last four trials used to calculate the fully adapted state. *R*^2^ values denote goodness of fit. Colour version available online

Since there was no interaction between visible block size and haptic size change on the scale of adaptation, the average rate of grasp adaptation was taken over all visible block sizes (40, 55, 70 mm) for increased and decreased conditions, and for each age group separately (Fig. 4a). The rate of adaptation was analysed using a repeated measures ANOVA with haptic size (increased, decreased) as the within subjects factor, and age group and whether participants noticed the manipulation as between subjects factors. A main effect of haptic size change was found [*F*(1, 44) = 12.191, *p* = 0.001, *ƞ*^2^ = 0.217]. Pairwise comparisons revealed that, on average, participants adapted more slowly to a decrease in the haptic block size (mean *T*_50%_ = 2.8 trials) than an increase (mean *T*_50%_ = 1.9 trials). A main effect of participant awareness of the manipulation was also found [*F*(1, 44) = 4.772, *p* = 0.034, *ƞ*^2^ = 0.098], with aware participants adapting faster (mean *T*_50%_ = 2.1 trials) than unaware participants (mean *T*_50%_ = 2.9 trials) (Fig. 4b). No further main effects or interactions were found (all *p* > 0.05).

### Rate of re-adaptation

The rate of re-adaptation was analysed in the same way as adaptation, which also revealed a main effect of haptic size change [*F*(1, 44) = 7.204, *p* = 0.010, *ƞ*^2^ = 0.141]. Pairwise comparisons showed that, on average, participants re-adapted faster from a decrease in the haptic block size (i.e. when the haptic block size increased; mean *T*_50%_ = 1.9 trials) compared to re-adapting from an increase in the haptic block size (i.e. when the haptic block size decreased; mean *T*_50%_ = 3.4 trials), thus reflecting a similar pattern to the adaptation period. No further main effects or interactions were found (all *p* > 0.05), including the main effect of awareness of the manipulation [*F*(1, 44) = 0.602, *p* = 0.442, *ƞ*^2^ = 0.013].

**Fig. 5:**
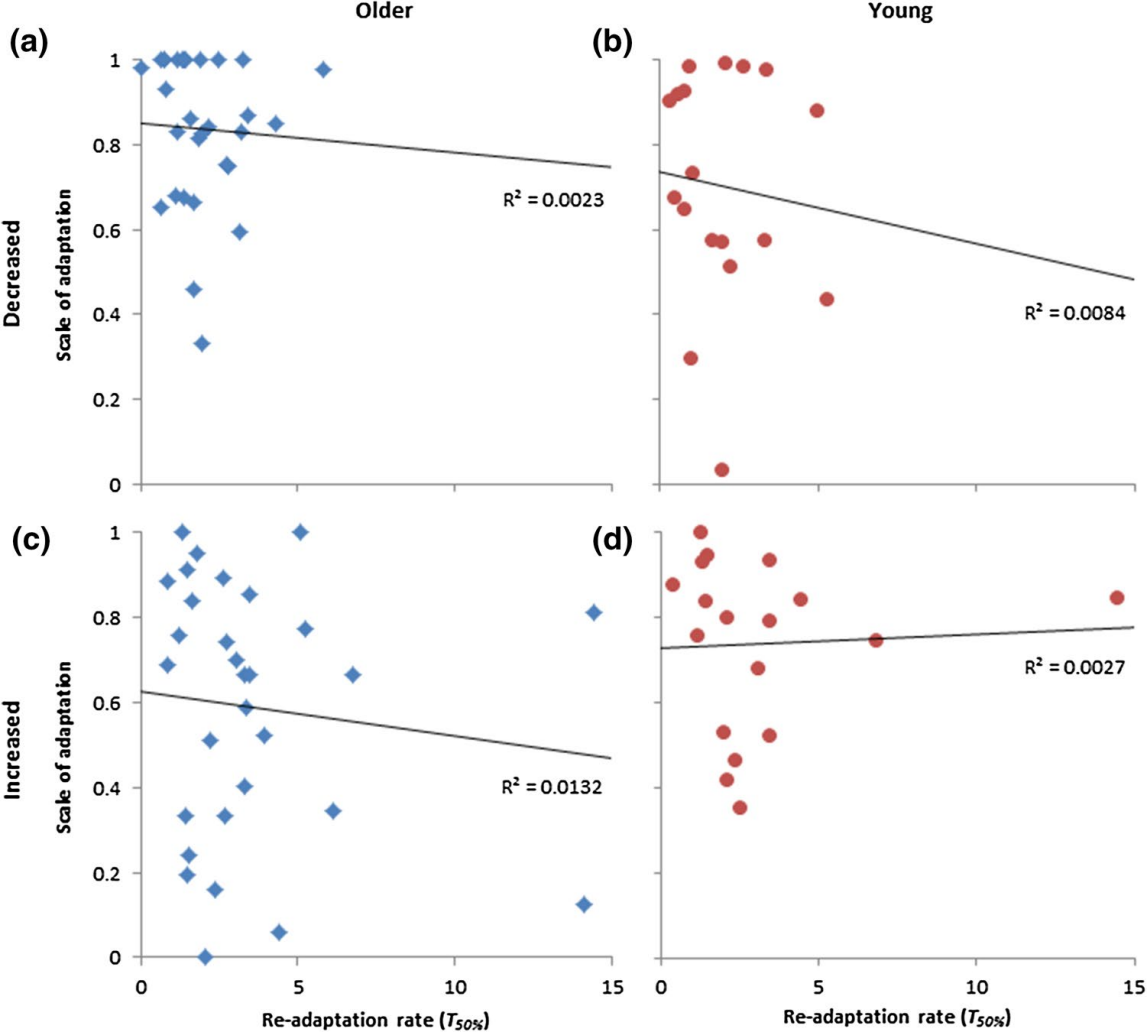
Correlations between the rate of re-adaptation and the scale of adaptation for increased size conditions for **a** older (blue diamonds) and **b** young (red circles) adults, and decreased size conditions for **c** older and **d** young adults. *R*^2^ values denote goodness of fit of the data to the trend lines (all non-significant, *p* > 0.05). Colour version available online

There were no significant correlations between the scale of adaptation and re-adaptation rates for increased or decreased haptic size conditions, for young or older adults (all − 0.12 < *r* < 0.05, *p* > 0.05), thus the size of the aftereffects was not related to the amount of adaptation that occurred (Fig. 5).

## Discussion

This experiment explored how young and older adults adapted their grasping behaviour in response to a visual–haptic size conflict. Contrary to predictions, older adults showed a similar scale of adaptation as young adults, although they adapted less when the haptic block increased in size compared to a decrease. For both age groups, an abrupt increase in the haptic block size lead to faster grasp adaptation compared to a decrease, and the time taken to adapt behaviour did not differ between age groups. Moreover, the size of aftereffects did not differ between young and older adults, with an abrupt increase in the haptic block size leading to faster grasp re-adaptation compared to a decrease for both age groups.

These findings appear to support some of the previous adaptation studies which have also shown similar levels of adaptation between young and older adults (Canavan et al. 1990; Etnier and Landers 1998; Roller et al. 2002; Bock and Schneider 2002; Buch et al. 2003; Cressman et al. 2010). There are several explanations for these results, which are not necessarily mutually exclusive. First is that the current sample of older adults had intact cognitive functioning, and thus strategic control mechanisms which contribute to the adaptation process were not affected by age-related cognitive decline (Bock 2005; Bock and Girgenrath 2006; Langan and Seidler 2011). However, further tests of cognitive functioning, such as spatial working memory (e.g. Anguera et al. 2011) and executive strategies (e.g. Huang et al. 2017), and their relation to grasp adaptation are required to elucidate this claim. A second explanation is that the conflict between visual and haptic cues was quite small (15 mm) meaning that there was little need for strategic control mechanisms (despite it being an abrupt change) and the conflict was easy to resolve, and therefore adaptation was robust to age-related cognitive decline. It may be beneficial to use a wider range of visual–haptic conflict sizes to determine whether age-related effects arise for larger sensory discrepancies. A third explanation is that, similarly to manual aiming tasks, the current grasping task (or grasping behaviour in general) was not operationally or cognitively complex enough to reveal age-related effects (Bruijn et al. 2012; King et al. 2013). For example, participants were not required to perform any secondary actions after the grasp, which have previously been shown to alter grasp kinematics with varying task complexity (Ansuini et al. 2006, 2008; Cicerale et al. 2014). Thus, a more goal-directed reach and grasp response and/or a secondary distractor task (e.g. Huang et al. 2017) might reveal age-related effects on grasp adaptation.

For the older adults only, we observed that a decrease in the unseen haptic block size leads to a greater scale of grasp adaptation compared to an increase in the haptic block size. Säfström and Edin (2004) claim that adaptation of MGA reflects the relative impact (or weighting) of haptic size information during the grasping movement. That is, if the adapted MGA more closely resembles the size change of the haptic block, then haptic information has been “up-weighted” over visual. In their experiment, it was suggested that an increase in haptic block size resulted in greater and faster haptic up-weighting compared to a decrease in haptic block size, since participants must rely more on haptic information to increase the clearance distance between the fingers and the block to avoid colliding with the edges. In the current experiment, it is possible that older adults weigh visual cues more highly (resulting in less grasp adaptation) in increased size conditions, which is consistent with previous research investigating sensory reweighting in older adults (Sundermier et al. 1996; Simoneau et al. 1999; Newell et al. 2011; Barrett et al. 2013). However, without determining how the senses were weighted prior to introducing the conflict, or how much visual weight may have changed by introducing the conflict, it is difficult to draw any firm conclusions. Furthermore, this would not explain why grasp adaptation (or “haptic up-weighting”) was reduced in increased size conditions compared to decreased size conditions in the current experiment.

An alternative explanation is that since older adults had slightly larger MGAs compared to young adults prior to introducing the visual–haptic conflict (non-significant), this would also mean that the fully adapted MGA in increased haptic conflict conditions would need to be larger in older adults to observe the same level of adaptation as young adults (see dashed black lines in Fig. 2). In addition, the block sizes in the current experiment were larger than that of the original experiment (i.e. up to 85 mm compared to 72.5 mm in Safström and Edin 2004), therefore older (and also young) adults’ grasp apertures may have reached their physical limit in increased size conditions, thus giving the appearance of less grasp adaptation. This seems likely given that aMGAs were similar for older and young adults in increased size conditions (see Fig. 2). Indeed, this could also explain the greater scale of grasp adaptation in decreased haptic size conditions for older adults, whereby it is easier to decrease their “overstretched” MGA in no-conflict conditions to the smaller blocks in conflict conditions. It is unclear why older adults would adopt a larger grasp aperture prior to introducing the conflict, but could be related to i) increased uncertainty in movement planning which could lead to safety behaviour to avoid a collision (Wing et al. 1986; Schlicht and Schrater 2007b, a), ii) a task requirement effect whereby less goal-directed movements may alter older adults’ grasp kinematics (Cicerale et al. 2014), iii) a grasping precision effect (Grabowski and Mason 2014) or iv) other anatomical/physiological changes to the ageing hand (Carmeli et al. 2003).

Also contrary to predictions, there was no difference in the rate of grasp adaptation between the age groups, despite the sudden introduction of the visual–haptic size conflict (Bock and Girgenrath 2006). However, participants who noticed the manipulation adapted their behaviour more quickly than those who did not. Awareness of the size change could arise from colliding with the edge of the block after it became larger, and could, therefore, explain some of the overall asymmetries in adaptation rate, with adaptation occurring more quickly when the block size increased compared to decreased to ensure adequate grasp clearance (Säfström and Edin 2004). Being consciously aware of the sudden size change would also mean that more cognitive processing (i.e. strategic control) was used to solve the discrepancy, and thus participants were able to adjust their grasp size more rapidly (Buch et al. 2003; Bock and Girgenrath 2006).

The scale of grasp adaptation was not affected by participants being aware of the visual–haptic conflict. As such, the internal model and strategic control mechanisms eventually reached a similar outcome for adapting behaviour, irrespective of age and being aware of the manipulation (Redding and Wallace 1996; Kawato and Wolpert 1998; Wolpert et al. 1998; Wolpert and Kawato 1998). In addition, there was no significant difference in the proportion of adults who noticed the size manipulation between the two age groups, nor were there any significant interactions between age group and awareness for the rate of adaptation. This suggests that strategic control mechanisms for grasp adaptation, including awareness, are not affected by cognitive decline in healthy ageing (Bock 2005; Bock and Girgenrath 2006; Anguera et al. 2011).

In line with predictions, the speed of re-adaptation (i.e. aftereffects) did not differ significantly between age groups. This is unsurprising given that the scale and rate of grasp adaptation was similar for both age groups, and that re-adaption is mostly governed by internal models which are less affected by age-related cognitive decline (Bock 2005; Bock and Girgenrath 2006). Similarly, this could also explain why there was no significant difference in the rate of re-adaptation between participants who were aware of the visual–haptic conflict and those who were not, since strategic control mechanisms (e.g. awareness) are less effective in the absence of sensorimotor conflict (Bock 2005; Bock and Girgenrath 2006). Note, however, that the scale of adaptation was not related to the strength of aftereffects, which could suggest that the mechanisms for grasp adaptation to a visual–haptic conflict (i.e. strategic control mechanisms) differ to those for grasp re-adaptation (i.e. internal models only), or that there is a large amount inter-individual variability (see Fig. 5). Alternatively, it could be that since the scale of grasp adaptation was limited by the physical capabilities of the hand (especially for increased size conditions), it is not possible to fully observe the relationship between adaptation and aftereffects.

## Conclusion

Whilst ageing might impair motor skills and sensorimotor adaptation for certain manual aiming tasks, the current findings suggest that healthy older adults show a comparable scale and rate of grasp adaptation as young adults. This suggests that age-related difficulties with daily activities involving prehensile actions cannot be explained by poorer grasp adaptation abilities. However, it would be interesting to test the current paradigm in older adults with some level of clinically significant cognitive decline (as opposed to general age-related cognitive decline) and/or general motor impairments (i.e. musculoskeletal issues or disease of the motor system), which could help to determine/disentangle the roles of cognitively demanding strategic control mechanisms and the physical capabilities of the hand in grasp coordination.
